# Pannexin-1 Deficiency Decreases Epileptic Activity in Mice

**DOI:** 10.3390/ijms21207510

**Published:** 2020-10-12

**Authors:** Mark S. Aquilino, Paige Whyte-Fagundes, Mark K. Lukewich, Liang Zhang, Berj L. Bardakjian, Georg R. Zoidl, Peter L. Carlen

**Affiliations:** 1 IBME, University of Toronto, 164 College Street, Rosebrugh Building, Room 407, Toronto, ON M5S 3G9, Canada; berj.bardakjian@utoronto.ca (B.L.B.); Peter.Carlen@uhnresearch.ca (P.L.C.); 2 Krembil Research Institute, University Health Network, 135 Nassau Street, Toronto, ON M5T 1M8, Canada; m.lukewich@mail.utoronto.ca (M.K.L.); Liang.Zhang@uhnresearch.ca (L.Z.); 3 Department of Biology, York University, 4700 Keele Street, Toronto, ON M5S 3G9, Canada; whytefp@yorku.ca (P.W.-F.); gzoidl@yorku.ca (G.R.Z.)

**Keywords:** panx1, seizure, epilepsy, PTZ, 4-AP, kindling

## Abstract

Objective: Pannexin-1 (Panx1) is suspected of having a critical role in modulating neuronal excitability and acute neurological insults. Herein, we assess the changes in behavioral and electrophysiological markers of excitability associated with Panx1 via three distinct models of epilepsy. Methods Control and Panx1 knockout C57Bl/6 mice of both sexes were monitored for their behavioral and electrographic responses to seizure-generating stimuli in three epilepsy models—(1) systemic injection of pentylenetetrazol, (2) acute electrical kindling of the hippocampus and (3) neocortical slice exposure to 4-aminopyridine. Phase-amplitude cross-frequency coupling was used to assess changes in an epileptogenic state resulting from Panx1 deletion. Results: Seizure activity was suppressed in Panx1 knockouts and by application of Panx1 channel blockers, Brilliant Blue-FCF and probenecid, across all epilepsy models. In response to pentylenetetrazol, WT mice spent a greater proportion of time experiencing severe (stage 6) seizures as compared to Panx1-deficient mice. Following electrical stimulation of the hippocampal CA3 region, Panx1 knockouts had significantly shorter evoked afterdischarges and were resistant to kindling. In response to 4-aminopyridine, neocortical field recordings in slices of Panx1 knockout mice showed reduced instances of electrographic seizure-like events. Cross-frequency coupling analysis of these field potentials highlighted a reduced coupling of excitatory delta–gamma and delta-HF rhythms in the Panx1 knockout. Significance: These results suggest that Panx1 plays a pivotal role in maintaining neuronal hyperexcitability in epilepsy models and that genetic or pharmacological targeting of Panx1 has anti-convulsant effects.

## 1. Introduction

The pannexin-1 (Panx1) protein forms a transmembrane channel with a vital, yet unresolved, role in modulating cellular hyperexcitability [1,2,3]. As Panx1 is highly expressed in the post-synaptic compartment of hippocampal and cortical pyramidal neurons, it is in a prime position for interacting with the molecular, structural, ionic and electrical changes in the synaptic cleft during active communication. The combination of function and position of Panx1 channels makes them a noteworthy contributor to the hyperexcitable dynamics in epilepsy.

What is known of Panx1 in epilepsy is that the protein becomes upregulated in association with seizures in both rodent and human tissue [4,5,6,7]. It has also been implicated in excitability dynamics through its involvement in modifying synaptic plasticity [8]. However, the immediate contribution of Panx1 to seizure-like activity is unclear, as the protein has been purported to both excite [9,10,11] or inhibit [12,13] seizure-like excitability across different models and even cell types [14].

Herein, we set out to assess changes in behavioral and electrophysiological markers of excitability associated with Panx1. We interrogate various brain regions through different models of epilepsy, focusing on analyzing local field potential recordings in mice with Panx1 channels blocked or genetically deleted. Changes in brain oscillations and how they couple with one another have been shown to be biomarkers of excitability. Therefore, we look to investigate changes in epileptiform rhythms and phase-amplitude coupling, such as theta, gamma and high frequency (HF) rhythms [15,16] as well as delta-HF [17] and delta-gamma [18,19] coupling.

We conclude that, across these three models of seizure induction, inhibition of Panx1 function promotes a seizure-resistant phenotype. Furthermore, we present evidence that pretreatment with Panx1 blocking agents, probenecid and Brilliant Blue-FCF, reduces seizure severity and prevents the onset of seizures.

## 2. Results

### 2.1. PTZ Injections

First, we investigated the systemic effect of convulsants on Panx1 deficient mice. Following the administration of a large dose (80 mg/kg) of PTZ [20,21], all 48 mice developed seizures of at least stage 5.

**Figure 1:**
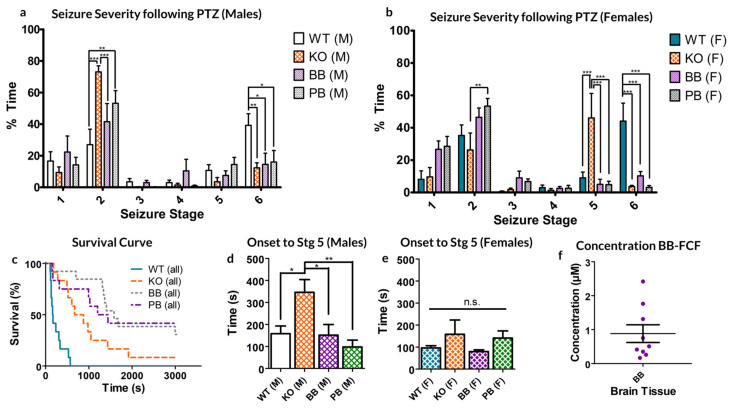
Response to 80 mg/kg pentylenetetrazol (PTZ) injection in each of the four animal groups, WT (normal pannexin-1 expression), KO (genetic knockout of pannexin-1) and WT-treated BB (pretreated with Brilliant Blue-FCF) or PB (pretreated with probenecid) mice. (**a**,**b**) male and female (respectively) mice demonstrated altered distributions of time spent manifesting behaviors of different seizure stages. (**c**) Survival curve of both sexes of animals following PTZ injection. (**d**,**e**) Average time to first stage 5 seizure. No significant differences exist between the two sexes of the same experimental group. KO Males took significantly longer to reach stage 5 seizures than any other group except KO females. (**f**) The average concentration of BB-FCF, as measured through spectrophotometry of homogenized brain tissue, was approximately 0.88 ± 0.26 µM. In all cases, * *p* < 0.05, ** *p* < 0.01, *** *p* < 0.001, Two-way ANOVA with Bonferroni post-hoc test.

Both male and female WT mice spent significantly more time manifesting Stage 6 seizures than their counterparts in any other group (see Figure 1a,b). This represents an overall decrease in the seizure severity for groups deficient in Panx1 activity, however, the extent of this reduced excitability varied between sexes.

Female KO animals spent significantly more time in stage 5 seizures than any other group. Of note, female mice in the KO groups spent significantly less time in Stage 2 than PB mice (*p* < 0.01 Bonferroni post-hoc test; see Figure 1a).

Male mice only showed significant differences in stage 2 seizures, where both KO and PB-treated mice spent significantly more time than their WT and BB counterparts (KO: *p* < 0.001, PB: *p* < 0.01 Bonferroni; See Figure 1b). Male KO mice also spent more time in this state as compared to the BB mice (*p <* 0.001, Bonferroni).

Comparing across sexes, the genetic knockout of Panx1 had differential effects. For males, the proportion of time spent in the milder seizure state 2 is dramatically increased, whereas in females, it is state 5 in which KO mice spend a disproportionately high percentage of their time.

Concerning the excitotoxic death, WT mice had the fastest mortality rate compared to any other animal group, with none of the animals surviving to the 30-min experimental limit (1800 s, see Figure 1c). Of those that died as a result of PTZ exposure, BB and KO mice survived significantly longer as compared to their WT counterparts (WT: median 165 s, KO: median 672 s, *p* = 0.0007, ANOVA). Of the KO group, one male survived to the experimental conclusion; of the BB group, three females survived; and of the PB group, four females and one male survived. No statistically significant source of variation exists between the two sexes of animals (*p* = 0.1294, Two-way ANOVA).

Finally, KO males took significantly longer to develop a seizure of at least stage 5 than every other group, other than KO females, to which the difference was not significant (see Figure 1d,e).

As far as we know, this is the first reported case of therapeutic effect achieved from injecting BB-FCF i.p. As such, the concentration of BB-FCF in the brain was measured via absorbency measures from homogenized brain samples and found to be approximately equivalent to a concentration of 0.88 *±* 0.26 µM (95% CI: 0.28–1.48 µM, see Figure 1e). This is above the previously reported IC50 of 0.27 µM BB-FCF on Panx1 [22].

### 2.2. Electrical Kindling of Hippocampal CA3 Region

Next, electrical kindling was applied directly to the CA3 region of the hippocampus in order to assess whether the altered propensity to seizure persisted at the level of local circuitry. Of the 12 WT animals (5 males, 7 females) that underwent electrical kindling, 7 successfully kindled to at least one stage 5 seizure (2 males, 5 females) and 2 others to stage 4 seizures (both females). Of the 10 knockout animals (6 males, 4 females), only one kindled to multiple stage 5 seizures (1 female).

**Figure 2:**
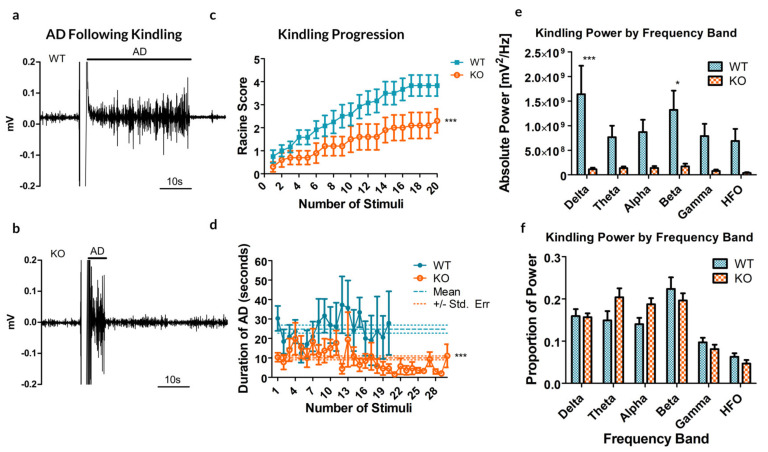
Results from the kindling of 12 Panx1 +/+ (WT) and 10 Panx1 −/− (KO) mice. (**a**,**b**) Sample recordings of the CA3 following electrical stimulation to induce kindling in WT (**a**) and KO (**b**) mice, demonstrating the difference in the afterdischarge (AD). These examples are reflective of the stimulus experienced by each mouse. (**c**) The maximal behavioral outcome experienced, as measured by the Racine score, averaged by group. A significant decrease in cumulative AD was observed in KO animals as compared to their WT counterparts. (**d**) The average duration of ADs following each stimulus showed no significant trial-dependent trends but strongly indicated a reduced AD duration in KO mice. Dashed lines show the mean AD duration and dotted lines represent the standard error of the mean (SEM). (**e**) Kindling power by frequency band. Significant differences are reported by band, between the WT and KO groups (**f**) Percent total power by frequency band. * *p* < 0.05, *** *p* < 0.001, Student’s *t*-test.

Remarkably, the rate of cumulative ipsilateral ADs between all trials was significantly reduced in the Panx1 null mice compared to controls (*p* = 0.0041, *t*-test, see Figure 2a–c). As for the duration of ADs following stimulation, a significant difference exists between the two groups, indicating that the average AD is shorter in the KO mice (10.02 *±* 1.01 s, compared to 24.88 *±* 2.11 s for WT; *p <* 0.0001, *t*-test, Figure 2d).

Analyzing the spectral power of the ADs revealed a significant increase in overall spectral power (see Figure 2e, *p* < 0.0001, Bonferroni post-test), specifically in the delta (*p <* 0.001) and beta (*p* = 0.01535) bands. These specific frequency bands in electrical kindling have previously been associated with the presence of stage 1–2 seizures and stage 4–5 seizures respectively [23].

### 2.3. Cortical Slices Exposed to 4-AP

To continue the investigation into altered electrophysiological activities at the local circuit level, we examined local field potentials in neocortical slices *in vitro*. Extracellular recording electrodes placed in layer II/III of neocortical slices recorded “spontaneous” or self-sustained population responses to the convulsant 4-AP.

**Figure 3:**
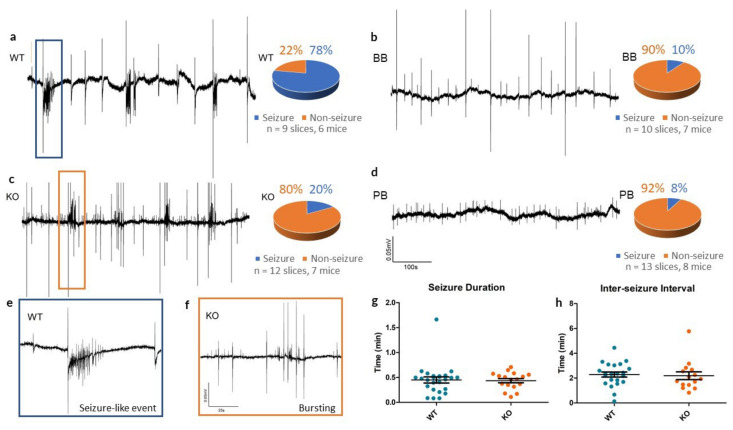
Seizure-like events in vitro in WT, KO, BB (Brilliant Blue-FCF-treated) and PB (probenecid-treated) groups. (**a**–**d**) Sample recordings of local field potentials from each of the four experimental groups, along with pie charts summarizing seizure incidence with 4-AP. Recordings from WT mice often had one or more seizure-like events (78%), whereas KO, BB and PB mice often had only small synchronous bursting events (20%, 10% and 8% respectively). All traces share the calibrations defined in (**d**). (**e**,**f**) High-energy activity in the WT and KO traces is zoomed in to reveal a SLE in the WT slice and bursting in the KO slice, indicative of the most common outcomes to 4-AP for each group. Both traces share the calibrations defined in (**f**). (**g,h**) Comparison of seizure duration and inter-seizure interval of SLEs in both WT and KO slices. Despite a reduced incidence, once initiated, KO SLEs remained similar to WT SLEs.

In slices from WT animals, 4-AP reliably produced SLEs (see Figure 3), whereas Panx1 inhibition resulted in rare epileptiform responses to 4-AP (see Table 1). In the KO group, traces from cortical slices exposed to 4-AP showed small population spikes (0.75 *±* 0.02 s in duration) but in only 2 of the 12 slices tested was there evidence of SLEs.

In those that did produce SLEs, there was no significant differences in the duration of events (WT: 0.45 ± 0.06 min, KO: 0.44 ± 0.04 min, *p* = 0.861, *t*-test) or in the inter-SLE interval (WT: 2.29 ± 0.20 min, KO: 2.19 ± 0.31 min, *p* = 0.794, *t*-test) between WT and KO groups. This suggests that Panx1 blockade may have a more pronounced role as an anti-epileptogenic - suppressing the mechanisms triggering seizure as opposed to lessening the severity of seizures themselves.

We also performed additional experiments to distinguish the direct contribution of Panx1 function from any developmental or compensatory effect arising from the genetic deletion of Panx1. Cortical slices from both BB and PB groups were incubated in each group’s respective Panx1 blocker for 10 min to ensure blockade before the concomitant addition of 4-AP to the perfusion media.

Extracellular field recordings from BB mice often contained small synchronous spiking events (1.63 ± 0.13 s in duration), similar to what was observed in the KO traces, however, they rarely exhibited SLEs. Across 10 4-AP experiments in slices of the BB group, only one case of seizure-like events occurred following pretreatment with 10 µM BB-FCF.

Similarly, across 13 experiments in the group pretreated with 500 µM probenecid, group PB, only one case of a SLE occurred. PB brain slices exhibited no noteworthy spontaneous activity.

#### Cross-Frequency Coupling In Vitro

**Figure 4:**
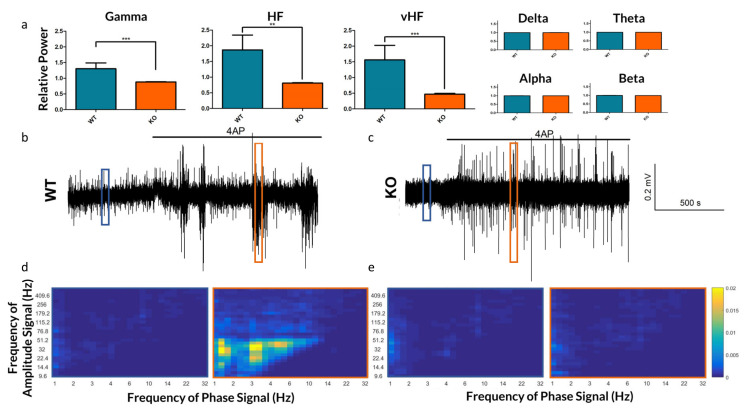
Frequency-based analysis of the extracellular recordings of Panx1 KO mice and their WT counterparts. (**a**) The relative increase by frequency band in neocortical field potentials after bath application of 4-AP. As 4-AP induces broadband increased spectral power, the relative increase in power of each band is normalized to the overall increase in spectral power. While changes in Delta through Beta bands were not significant, the increase in spectral power in Gamma (*p* = 0.0009), HF (*p* = 0.0011) and vHF (*p* = 0.0008) bands were significantly lower in Panx1 deficient animals following 4-AP. (**b**,**c**) Traces of WT and KO extracellular recordings under 4-AP. (**d**,**e**) Phase-amplitude coupling comodulograms of 10-s windows during baseline (blue) and 4-AP (orange) conditions, as highlighted in subfigures b and c respectively, highlighting periods of enhanced PAC in the WT 4-AP response which are absent in the KO. ** *p* < 0.01, *** *p* < 0.001.

We further assessed changes in frequency dynamics observed in vitro by analyzing various frequency bands (see Figure 4). As 4-AP induced a broadband increase in spectral power, we normalized the change of power across each frequency band to the overall increase in spectral power. This highlighted a diminished effect of 4-AP on Gamma, HF and vHF frequency bands in the KO, each of which are frequencies associated with epileptic signaling [15,24].

In addition to differences in the raw frequency power, we explored the role of Panx1 in mediating frequency synchronization. PAC strengths were computed for 9 WT and 12 KO slices exposed to 4-AP. WT slices demonstrated strong PAC during SLEs, whereas KO slices had very little (see Figure 4d,e).

**Figure 5:**
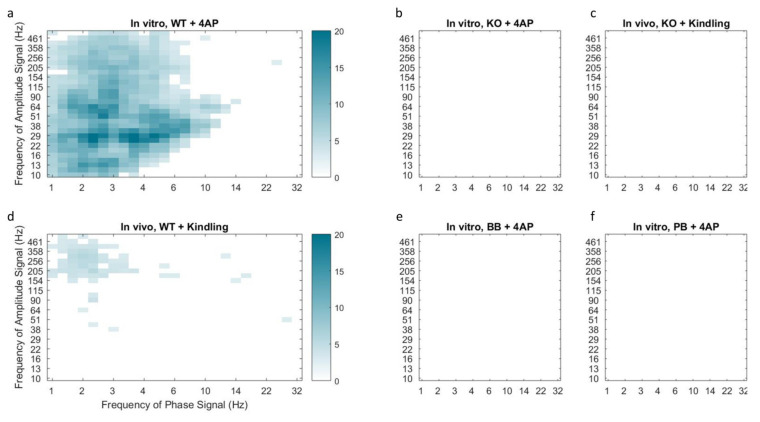
Median phase-amplitude cross frequency coupling comodulograms for the in vitro 4-AP slice model (**a**,**b**,**e**,**f**) and in vivo kindling model (**c**,**d**) following z-score normalization to baseline conditions. Cortical slices from mice in the KO group demonstrated reduced phase-amplitude coupling following incubation with 4-AP (**b**) or electrical kindling (**c**) as compared to WT counterparts (**a** and **d** respectively). Coupling of delta phase to HF amplitude was observed in both models of hyper-excitability in control animals but was absent in Panx1 deficient mice, including those pretreated with Panx1 blockers BB-FCF (**e**) and probenecid (**f**). This finding is indicative of reduced epileptiform activity in the absence of functional Panx1.

Quantifying these results, we observed that the median PAC of KO slices did not exceed the threshold of 3 standard deviations above the baseline mean, whereas WT slices under 4-AP demonstrated several frequency pairings of increased coupling (see Figure 5a,b), of which Delta-Gamma [25,26] and Delta-HF [17] PAC are relevant to epilepsy. These results may implicate Panx1 in the coupling of certain electrographic rhythms, as has previously been implied for other members of the gap junction family [27,28].

A similar absence of coupling was observed in mice pretreated with Panx1 blockers (see Figure 5e,f). Furthermore, WT mice that underwent electrical kindling demonstrated increased Delta-HF coupling which was once again absent in the KO group (see Figure 5c,d). Taken together, these findings suggest that Panx1 activity contributes to the presence of the Delta-HF cross-frequency coupling associated with hyperexcitability.

## 3. Discussion

Three distinct models of epilepsy presented here corroborate a significant pro-epileptic effect of the Panx1 channel. When Panx1 was blocked through chemical agents or was genetically deleted, both cortical and hippocampal tissue demonstrated reduced epileptiform activity following a convulsant insult. Acute kindling and in vitro trials suggested that Panx1 may play a role in epileptogenesis, as we observed the greatest impact of Panx1 action on the incidence of SLEs, while their duration and inter-SLE intervals were unaltered once initiated. Following acute injection of PTZ however, we did not observe an absence of SLEs but instead we saw reduced seizure severity. These results support a differential role of Panx1 in modulating excitability based on the mechanism of seizure induction.

Through systemic injection of a high dose of PTZ, both seizure severity and ictal-induced death were found to be significantly reduced through Panx1 KO or pharmacological blockade. In the case of Panx1 deletion, the progression to at least a stage 5 seizure was also reduced in males. To our knowledge, this is also the first demonstration of Brilliant Blue-FCF being delivered systemically and protecting against the effects of a convulsant chemical. As the presence of BB-FCF in the brain was confirmed through spectrophotometry, BB-FCF likely involves a crossing or interaction with the blood-brain barrier through unknown mechanisms.

When focusing on the local hippocampal circuitry, performing a paradigm of rapid electrical kindling in vivo highlighted a reduced propensity to generate ADs in Panx1 KO animals. This important phenotype resulted in a dramatically reduced incidence of SLEs as compared to Panx1 expressing animals. Most KO mice did not manifest any behavioral or electrographic signs of severe (stage 4/5) seizure.

Finally, populations of neurons within neocortical layers II/III demonstrated reduced susceptibility to 4-AP seizure-like events in a concentration-independent manner when Panx1 was functionally absent. This supports previously reported positive correlations between Panx1 and seizure activity [4,6].

### 3.1. Interpretation of Changes in CFC

Alterations in several key frequency pairings are indicative of altered communication in the Panx1 deficient mouse group. Delta-HF rhythms, in particular, have long been associated with severe epileptic responses and were notably absent in the 4-AP response.

It is important to remember that causation is unknown; these rhythms may be reflective of enhanced excitability, perhaps predisposing to SLE or reflective of the SLE activity directly. Either way, these results suggest that Panx1 may play a role in the PAC of various rhythms in the neocortex, including rhythms correlated with SLEs and epileptiform activity.

### 3.2. Anti-Epileptic Potential of Panx1 Therapeutics

As demonstrated by the systemic administration of BB-FCF, a definite anti-convulsant phenotype emerges from Panx1 blockade. This phenotype is recapitulated through in vitro application of the blocker on acute slices exposed to 4-aminopyridine, as well as with complete genetic knockouts of Panx1. This supports recent findings that Panx1 may be an effective target as an anti-convulsant or perhaps even in preventing epileptogenesis [10,11].

However, ongoing studies are needed in order to better characterize the electrophysiological and biophysical effects of Panx1 channel blockade on neural excitability. Of additional note, a key hurdle in evaluating Panx1 as a candidate for personalized medicine is the possible sex-specific consequences of Panx1 channel blockade or genetic deletion.

### 3.3. Sex-Specific Differences in Seizure Severity of Panx1 Knockouts

Following PTZ injection, we observed a significant increase in the proportion of time spent in seizure state 2 for KO males relative to all other groups. In contrast, KO females had a similarly disproportionate increase in the time spent in the more severe state 5.

The only mouse from the KO group to have successfully kindled to multiple stage 5 seizures following electrical kindling was a female. To what extent these observations are related to the estrous cycle of the mice used in these experiments is unknown, though hormonal changes are known to have significant effects on the susceptibility to seizure [29].

## 4. Materials and Methods

### 4.1. Experimental Design

Experimental Groups

C57Bl/6 mice (Charles River Laboratory) and Panx1 knockouts of the same background (CMV-Cre/Panx1fl/fl, courtesy of Dr. Valery Shestopalov, University of Miami [30]) were included in this study. Mice of both sexes and of various ages (detailed below by experiment) were considered. The mice were separated into four recording groups based on experimental conditions and Panx1 function.

1. WT: Unaltered Panx1 expression.
2. BB: Pretreated with BB-FCF to induce acute pharmacological blockade of Panx1.
3. PB: Pretreated with probenecid to induce acute pharmacological blockade of Panx1.
4. KO: Genetic knockout of Panx1.

All mice were maintained in a vivarium with a 12-h day/night cycle. All experimental procedures described below were reviewed and approved by the Animal Care Committee of the University Health Network, in accordance with the Guidelines of the Canadian Council on Animal Care.

### 4.2. Pharmacological Agents

FD&C Blue No. 1, also known as Brilliant Blue-FCF (BB-FCF, in vitro, 10 µM in the bath perfusate and in vivo, 60 mg/kg injected i.p. Dosages were selected based on the effective dose-response curve [22] and previous trials with the structural cousin, BBG [31].) and probenecid (PB, in vitro, 500 µM in the bath perfusate [32] and in vivo, 250 mg/kg injected i.p. [33]) were used as Panx1 blockers. Pentylenetetrazol (PTZ, in vivo, 80 mg/kg injected i.p.) and 4-aminopyridine (4-AP, in vitro, 100 µM in the bath perfusate) were used as convulsants to induce seizure-like events (SLEs).

#### 4.2.1. In Vivo PTZ Injection

Forty-eight adult mice (aged 4–6 months, 12 in each of the four groups, even split of sexes) were injected with 80 mg/kg PTZ into the peritoneal cavity (i.p.). Each animal was monitored for a maximum of three hours after injection, with the first 30 min under video recording. All behavioral markers of seizure states were tracked throughout the first 30 min following injection and were independently confirmed with video footage. No pretreatment was performed for the animals of groups WT or KO. For the animals of group BB, 60 mg/kg BB-FCF was administered i.p. five hours prior to the injection of PTZ. 250 mg/kg probenecid was delivered i.p. three hours prior for PB mice.

BB-FCF has not yet been conclusively shown to permeate across the blood-brain barrier, although the structurally similar compound BBG has been [34]. To confirm the presence of BB-FCF in the brains of mice following injection, brain tissue was extracted from each BB mouse immediately after death and sonicated for 2 min in a buffer of phosphate-buffered saline. The solution was then spun in a centrifuge for 20 min at 14,500 RPM at 4 oC. In a SPECTROstar Nano absorbance plate reader (BMG Labtech), 50 mL of each supernatant was run in duplicate at an excitation of 628 nm. BB samples were controlled against four samples prepared from WT mice immediately following the experiment.

#### 4.2.2. In Vivo Electrical Kindling of Hippocampus

A paradigm of rapid kindling [35] was used for kindling the hippocampus of 12 WT and 10 KO mice aged 25–35 days. All animals were anesthetized with isoflurane (5%) and implanted with five electrodes (a bipolar pair in each hippocampus and one reference electrode in the cortex) as previously described [36]. Following implantation, mice were given three days to recover before stimulation.

Electrical stimuli were applied unilaterally to the CA3 to induce electrical kindling. A stimulus train of monophasic square waveforms at 60 Hz, with a pulse duration of 0.5 ms and current intensities of 10–100 µA, was generated by a Grass stimulator and delivered through an S88 Grass isolation unit in 2 s trials.

The minimum stimulus threshold needed to induce an afterdischarge (AD; a synchronous bursting event of a population of neurons following the cessation of the driving stimulus) of at least 5 s was determined via an ascending series of stimulus trains, applied in increasing 10 µA steps every 30 min. A suprathreshold stimulus (2 s, 60 Hz, 110% of the minimum AD threshold) was applied every 15 to 30 min to induce electrical kindling. Once the stimulus was delivered, the bipolar stimulating electrodes were immediately switched to a recording mode to record the ipsilateral response.

Signals from each bipolar pair of electrodes were amplified 1000 times using external amplifiers (Model-300, AM Systems Inc.) and then digitized (Digidata 1400/1500, Molecular Systems). Each recorded signal was analyzed for the presence of an AD following stimulation.

Stimuli were applied 15 times daily to either (1) a maximum of 30 trials or (2) until two or more stage-5 seizures were observed in the same animal (seizure-state classification discussed below). Concurrent electrographic and video recordings were used to verify the animals’ responses following each stimulus.

#### 4.2.3. In Vitro Field Potentials under 4-AP

Mice aged P14–25 days from all four groups were anesthetized with isoflurane (5%) and quickly decapitated. Each brain was dissected and cut into 500 µm thick coronal slices with a Leica vibratome (1200 VT) using an ice-cold oxygenated dissection solution (composition (in mM)—sucrose (248), KCl (2), MgSO4 (3), CaCl2 (1), NaHCO3 (26), NaH2PO4 (1.25), d-glucose (10). These slices were incubated in carbonated artificial cerebrospinal fluid (aCSF, composition (in mM): NaCl (123), KCl (3.5), MgSO4 (2), CaCl2 (1.5), NaHCO3 (25), NaH2PO4 (1.2), d-glucose (10) as previously described [37]. These slices were then transferred and pinned to a submerged mesh surface to permit continuous perfusion of carbogenated 35 oC aCSF across the slice at a flow rate of 10 mL/min.

All slice electrophysiology recordings were performed with a Multiclamp 700B (Molecular Devices) amplifier and recorded with a Digidata 1322 (Molecular Devices) digitizer.

### 4.3. Identification and Classification of SLEs

As perturbations into the function of Panx1 were expected to result in changes of the typical SLEs, it was necessary to ensure that behavioral and electrographic activity of Panx1 impaired animals could be correctly identified as an SLE if one were to occur. As such, multiple classification metrics were used to detect and classify SLEs:

#### 4.3.1. In Vitro Electrographic Classifier

Electrographic SLEs are typically defined by time-series features [38], such as duration and shape but can also be distinguished from intra-SLE activity by frequency-based features, such as an elevated theta rhythm and high frequency spiking [15,39]. As such, three methods were used to identify SLEs from extracellular field potentials: first, a visual screen using time-based features; second, a classifier trained on 4-AP-specific ictal events using time and frequency domain features; and finally, a custom linear classifier was designed for separating and assessing high theta events.

For visual screening, electrographic recordings were contrasted against classical 4-AP induced SLEs. In vitro, manifest in local field potentials as an initial bursting period, followed by a tonic episode and clonic ADs, all carried on top of a slow < 1 Hz positive current [40]. SLEs were defined as having a duration of *≥* 5 s and an amplitude of at least twice the baseline signal.

An automated Matlab-based classifier, using training samples from 4-AP-induced cortical SLEs, was used to identify potential SLEs based on both time and frequency domain components [41].

Finally, a custom linear classifier, similar to those previously described for the classification of EEG signals [42,43], was used to sort high-theta electrographic events from the Panx1-impaired animals into seizure or non-seizure states. In addition to the complete frequency-based and time-based feature set noted by Reference [42] which includes energy, entropy, standard deviation, mean, maximum, minimum, inter-quartile range and mean absolute deviation, two additional frequency-based features were added [44,45,46]:

1. A modulation index of cross-frequency coupling between delta (1–4 Hz) and gamma/HF (60–100 Hz) bands.
2. A percent of total spectral power in the range of high-frequency bands (HF, 100–200 Hz).

Within the extracellular recordings, events of variable duration were automatically identified based on theta frequency (4–8 Hz) power greater than twice the average of a one-minute baseline. Once a high-theta event was identified, frequency information was extracted by performing a Morlet complex wavelet transform (using the basis function cmor6–0.8125 from Matlab R2016a). Following feature extraction, a subsequent analysis in a linear discriminant classification between two states was performed (via Matlab R2016a function “classify”). In order to provide a training set for the linear classifier, all high-theta events in control animals exposed to 4-AP were used (including events defined as SLEs).

#### 4.3.2. In Vivo Behavioral Classifier

Video recordings were taken during the induction of all SLEs. For in vivo PTZ administration, a 6-point intensity scale for generalized PTZ seizure severity was used [47]. The following is a list of each stage with some of the predominant indicators of seizure behavior within each numbered stage:

1. Behavioral freezing;
2. Head clonus and facial jerking;
3. Neck jerks and lordotic posture;
4. Clonic seizure in sitting position;
5. Clonic or clonic/tonic seizures on belly or pure tonic seizure;
6. Clonic or clonic/tonic seizures on side or wild jumping;

For in vivo electrical kindling, a modified Racine scale, as discussed by Pinel et al. [48] was used to classify behavioral changes along an 8-point scale.

1. Mouth or facial movements;
2. Head nodding;
3. Contralateral forelimb clonus;
4. Symmetrical forelimb clonus with rearing;
5. Rearing and falling;
6. Forelimb clonus with multiple rearing and falling;
7. Wild running, jumping
8. Tonic posture and climbing

#### 4.3.3. Analysis of Phase-Amplitude Cross-Frequency Coupling

Local field recordings were processed in 10-s windows with a Morlet complex wavelet transform (using the basis function cmor6-0.8125 from Matlab R2016a) to extract phase and amplitude information of frequencies between 1–500 Hz. The strength of phase-amplitude coupling (PAC) between low-frequency phase information and high-frequency amplitude was measured using the method by Tort et al. [49]. These strengths were then clustered into groups based on frequency bands.

The frequency bands were delineated as follows: Delta (1–4 Hz), Theta (4–8 Hz), Alpha (8–13 Hz), Beta (13–30 Hz), Gamma (30–80 Hz), High Frequency (HF, 80–120 Hz), Very High Frequency (vHF, 120–500 Hz). For recordings under the 4-AP condition, unless no SLEs were detected, only windows classified as SLEs by both digital classifiers were selected for analysis. The median PAC of each 10-s window under 4-AP was z-scored relative to windows taken from its baseline and thresholded at 6 standard deviations above baseline.

### 4.4. Electrophysiological and Statistical Analysis

Analysis of electrophysiological recordings was performed in Matlab R2016a (Mathworks). All statistical testing was performed in Matlab R2016a (Mathworks) and Prism (GraphPad). Values are reported as mean ± standard error of the mean (SEM, where *n* = number of trials) unless otherwise stated. A one- or two-way ANOVA was performed, followed by Bonferroni’s or Dunnett’s post-hoc tests. In testing the significance of changed incidence of SLEs, a Fisher’s Exact test was used with a confidence interval (CI) of 95%.

## 5. Conclusions

Whether through systemic injection of PTZ, local electrical kindling or focal application of 4-AP, Panx1 impairment has been shown to play an important role in modulating behavioral and electrophysiological markers of neuronal excitability. This work highlights the complex yet vital connection between Panx1 and seizures and underscores the potential therapeutic value of targeting Panx1 in the central nervous system to prevent SLEs.

Ongoing efforts are needed to characterize this channel’s fundamental function, including elucidating a model that places Panx1 mediated ATP release in the synaptic cleft as a central player in the generation of SLEs. Furthermore, a pattern of sex-specific differences in functional Panx1 impairment requires ongoing investigation as it may be critical to the clinical relevance of Panx1 as an effective therapeutic target for epilepsy.

## Figures and Tables

**Table 1:** Incidence of seizure-like events (SLEs) in vitro following perfusion of cortical slices with 100 µM 4-AP. 12 slices from 7 KO mice had fewer 4-AP-induced SLEs than in the 9 slices from 6 of their WT counterparts (*p* = 0.0051, Chi-squared). Mice treated with Brilliant Blue-FCF (7 mice, 10 slices) or probenecid (8 mice, 13 slices) before 4-AP exposure also had fewer instances of at least one SLE as compared to untreated slices of WT mice (BB *p* = 0.0028; PB *p* = 0.0008, Chi-squared).

Type	Total Number	SLE-Positive	SLE-Negative	Percentage	${\tilde{\chi }}^{2}$ *p*
WT	9	7	2	78	-
KO	12	2	10	20	0.0051
BB	10	1	9	10	0.0028
PB	13	1	12	8	0.0008
ALL	-	-	-	-	<0.0001
